# Phylogenetic structure of body shape in a diverse inland ichthyofauna

**DOI:** 10.1038/s41598-023-48086-5

**Published:** 2023-11-25

**Authors:** Kevin T. Torgersen, Bradley J. Bouton, Alyx R. Hebert, Noah J. Kleyla, Xavier Plasencia, Garrett L. Rolfe, Victor A. Tagliacollo, James S. Albert

**Affiliations:** 1 Department of Biology, University of Louisiana, Lafayette, USA; 2 Federal University of Uberlândia, Uberlândia, Minas Gerais 38400-902 Brazil

**Keywords:** Evolution, Ichthyology

## Abstract

Body shape is a fundamental metric of animal diversity affecting critical behavioral and ecological dynamics and conservation status, yet previously available methods capture only a fraction of total body-shape variance. Here we use structure-from-motion (SFM) 3D photogrammetry to generate digital 3D models of adult fishes from the Lower Mississippi Basin, one of the most diverse temperate-zone freshwater faunas on Earth, and 3D geometric morphometrics to capture morphologically distinct shape variables, interpreting principal components as growth fields. The mean body shape in this fauna resembles plesiomorphic teleost fishes, and the major dimensions of body-shape disparity are similar to those of other fish faunas worldwide. Major patterns of body-shape disparity are structured by phylogeny, with nested clades occupying distinct portions of the morphospace, most of the morphospace occupied by multiple distinct clades, and one clade (Acanthomorpha) accounting for over half of the total body shape variance. In contrast to previous studies, variance in body depth (59.4%) structures overall body-shape disparity more than does length (31.1%), while width accounts for a non-trivial (9.5%) amount of the total body-shape disparity.

## Introduction

Body shape is a fundamental metric of functional diversity among taxa across the tree of life and among biotas across environmental and geographic gradients^1–4^. For aquatic animals, adult body shape and size strongly affect physiological and behavioral performances^5–7^ and these attributes are excellent predictors of many ecological and life history traits, evolutionary patterns, and conservation threats^8–11^.

Fishes represent excellent materials for the study of how measures of whole-body phenotypes, like body shape and size, affect ecological dynamics, spatial distributions, and conservation decisions^12–16^. The evolution and interspecific disparity of body shapes among fishes has been studied in a variety of faunas, including tropical reefs^17^, tropical freshwaters^18^, the deep sea^19^, and paleofaunas in deep time^20,21^.

**Figure 1:**
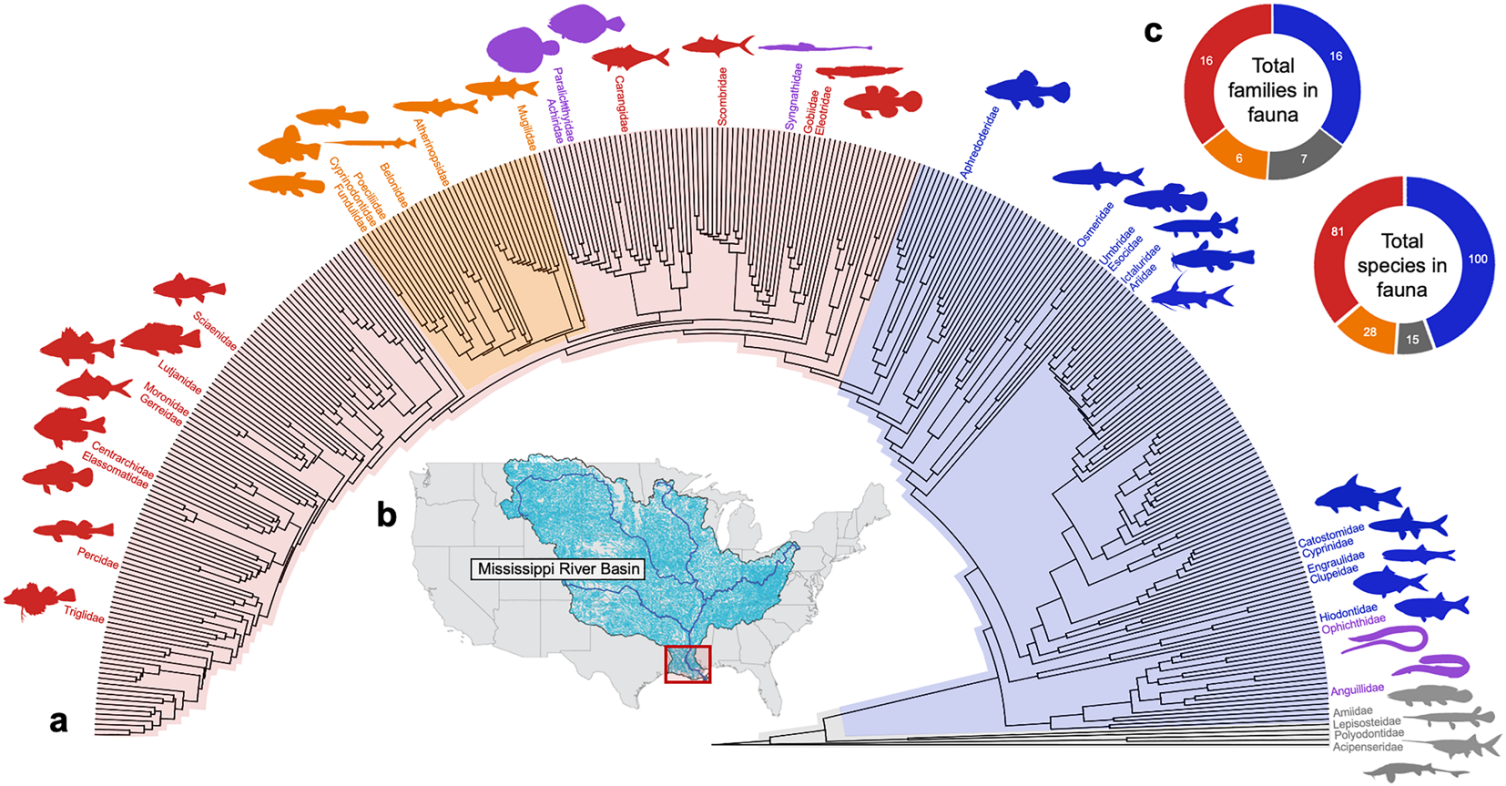
Phylogenetic diversity of the LMB fish fauna. (**a**) Time-calibrated phylogeny of extant ray-finned fishes (Actinopterygii), with family names and colored silhouettes illustrating 40 of 490 (8.1%) actinopterygian families represented in the LMB fish fauna. Colored polygons represent nested clades used in the analysis of morphospace occupancy: gray, non-teleosts; blue, non-acanthomorph teleosts, red, non-ovalentarian acanthomorphs; orange, ovalentarian acanthomorphs; purple, clades excluded from analysis due to lack of homologous landmarks. Colored silhouettes were generated from images of actual fish specimens from the LMB. (**b**) Map of Mississippi River basin with LMB study area highlighted in red box. (**c**) Species and family composition of the four assemblages within the LMB fish fauna. Taxonomic names follow accepted conventions for the field^109^.

The Lower Mississippi Basin (LMB) is one of the most phylogenetically diverse and phenotypically-disparate temperate inland fish faunas on Earth (Fig. 1)^14,22–24^, with at least 245 fish species assigned to over 40 families^25^. The LMB is a global hotspot of freshwater fish biodiversity^26^, displaying a wide range of phenotypes, including differing sizes, shapes, life-history strategies, and broad ecological and functional disparities that include both primary and secondary freshwater fishes and euryhaline species, with a lengthy geological history extending back to the Cretaceous Period (ca. 145–66 Ma). The fauna is widely known for its many relictual taxa who formerly had more geographically widespread or even global distributions (e.g., chondrostean sturgeons and paddlefishes, holostean gars and bowfins, osteoglossiform mooneyes and goldeyes)^27,28^. Taxa of the LMB fauna are derived from a wide range of phylogenetic and geographic sources including Eurasian freshwaters (e.g., esocid pikes; umbrid mudminnows; catostomid suckers; cyprinid minnows; leuscisid shiners; ictalurid catfishes; percid walleyes and darters), Central American freshwaters (e.g., poeciliid guppies)^29,30^, and marine waters (e.g., percopsid and amblyopsid troutperches, pirate perches and cavefishes; centrarchid sunfishes). The LMB fauna is also populated by numerous marine-derived taxa of relatively recent (i.e. Pleistocene, Holocene) phylogenetic origin (e.g., mugilid mullets; syngnathid pipefishes; sciaenid drums; pleuronectiform flatfishes; etc.). In addition to these natural attributes, the LMB is also among the most well-studied inland freshwater faunas on Earth^31–34^, and therefore represents a natural target for studies on the evolution and disparity of fish phenotypes^35^. Given, these considerations, the LMB is an excellent candidate for a study of body-shape evolution across an entire fauna.

The quantitative assessment of body-shape disparity among taxa and regions is a rapidly developing area of research^21,36–42^. Linear measurements of certain ecologically-relevant traits (e.g., mouth width, eye size, head and body length and depth) have long been used to assess overall body shape in fishes among species of a faunal assemblage^43,44^ or clade^45,46^. Point-to-point measurements are relatively easy to assess from freshly collected or preserved specimens, often without concern for variable specimen condition or other preservation artifacts (e.g., axial bending or variable mouth, opercle, or fin positions)^8,47,48^. However, linear measurements represent only a tiny fraction of the total information represented by the overall body shape of an organism. Further, a morphospace constructed from linear measurements is non-metric when it is composed of variables assessed using different scales, meaning the units of multivariate distance are undefinable and not comparable^49,50^.

The study of biological shape disparity was advanced by geometric morphometrics (GM) using Cartesian landmark coordinates to capture morphologically distinct shape variables^51–53^. GM allows investigators to assess shape variance while retaining the geometry of landmark points used to establish the homology of body regions^54–56^. A morphospace defined by GM is metric, meaning changes in all directions are measured in the same Procrustes shape units^57^. In the non-metric space of linear measurements, disparity of the axes is arbitrary, whereas the axes of a GM morphospace are scaled from the translation, rotation, and scaling steps of the Procrustes superimposition^58^. The use of ratios and ad hoc combinations of spatially unrelated linear measures is therefore biased regarding geometrical shape information^59^.

Studies using two-dimensional (2D) GM in species-rich fish faunas (Table 1) and individual clades^18,60–63^ usually find differences in axial (i.e. anterior–posterior) length and dorso-ventral depth as the most prominent axes of body-shape variance (i.e. PC1 and PC2). Yet 2D GM studies are blind to variance in body width, a prominent aspect of body shape in fishes with dorsoventral body compression (e.g., myliobatiform stingrays, siluriform catfishes, balitorid hillstream loaches, lophiiform goosefishes), and groups with a laterally-expanded body shape (e.g., tetraodontiform boxfishes and pufferfishes; lophiiform frogfishes and anglerfishes). Several aspects of body width have important functional consequences in fishes, for example in foraging and prey consumption (mouth width)^64^, respiration (gill surface area and interopercular distance)^65^, sensory reception (interorbital and internarial distances)^66^, and locomotion (maximum cross sectional area)^67^.

**Table 1:** Summary of published studies on body-shape disparity in fish faunas.

Biomes	Diversity	Methods	PC1	PC2	PC3	Refereces
Marine	423 fossil spp.	2D GM, PCA	AP length, DV depth	Median fins	DF length	21
Marine reefs	2939 spp.,	2D GM, PCA	AP length, DV depth	Post-coelomic length	Coelomic AP&DV	36,37
	56 families					
Marine reefs	1530 spp.,	LM, PCA	AP length, DV depth	DV depth	NA	41
	111 families					
Marine reefs	3344 spp.,	LM, PCA	AP length, DV depth	DV depth	NA	39
	268 families					
Marine deep sea	3033 spp.,	LM, PCA	AP length, DV depth	DV depth	NA	40
Marine & freshwater	263 families	LM, PCA	AP length, DV depth	NA	NA	95
Marine	2295 spp.	LM, PCA	AP length, DV depth	Width	NA	42
				NA	NA	113
Marine & freshwater		LM, PCA	AP length DV depth			
	5610 spp.					

Studies arranged by year of publication. Abbreviations: LM, linear measurements, GM, geometric morphometrics; PCA, principal components analysis; AP, anterior–posterior; DV, dorso-ventral; DF, dorsal fin; NA, data not provided. Note many of the LM studies are based on overlapping datasets.

In the last decade, three-dimensional (3D) geometric morphometrics have been used with microcomputed tomography (µCT) scan data to attempt a more complete evaluation of 3D body shape compared to previous methods. However, the acquisition of µCT data is both prohibitively costly and time consuming to do on a large scale. Recently, methods to create 3D models that accurately represent the external body shape of biological specimens using structure-from-motion (SFM) 3D photogrammetry have become increasingly inexpensive and user-friendly^68^. 3D photogrammetry provides the community a publicly available corpus of photorealistic 3D digital models that can be used in a wide variety of contexts and purposes including biomechanics, functional morphology, systematics, ecophysiology, education, and public outreach.

Here we use recently-developed photogrammetric methods to generate 3D digital models of adult body shape of fishes in the LMB fauna. We use GM of 3D landmark coordinates representing homologous point locations on the model surfaces to study interspecific shape differences, and use Principal Components Analysis (PCA) to generate geometrically independent deformations of whole-body shape change^69^. This work began as an undergraduate class project in an upper-level ichthyology course taught by the senior author in Fall 2022, and highlights what we found to be an excellent way to engage undergraduate students in meaningful specimen-based research activities. The aim of this study is to quantitatively assess the phylogenetic structure of 3D body shape among members of the LMB fish fauna, and contributes to understanding the role of body shape disparity in the accumulation of biodiversity at regional scales^18,37,70,71^.

## Results

**Figure 2:**
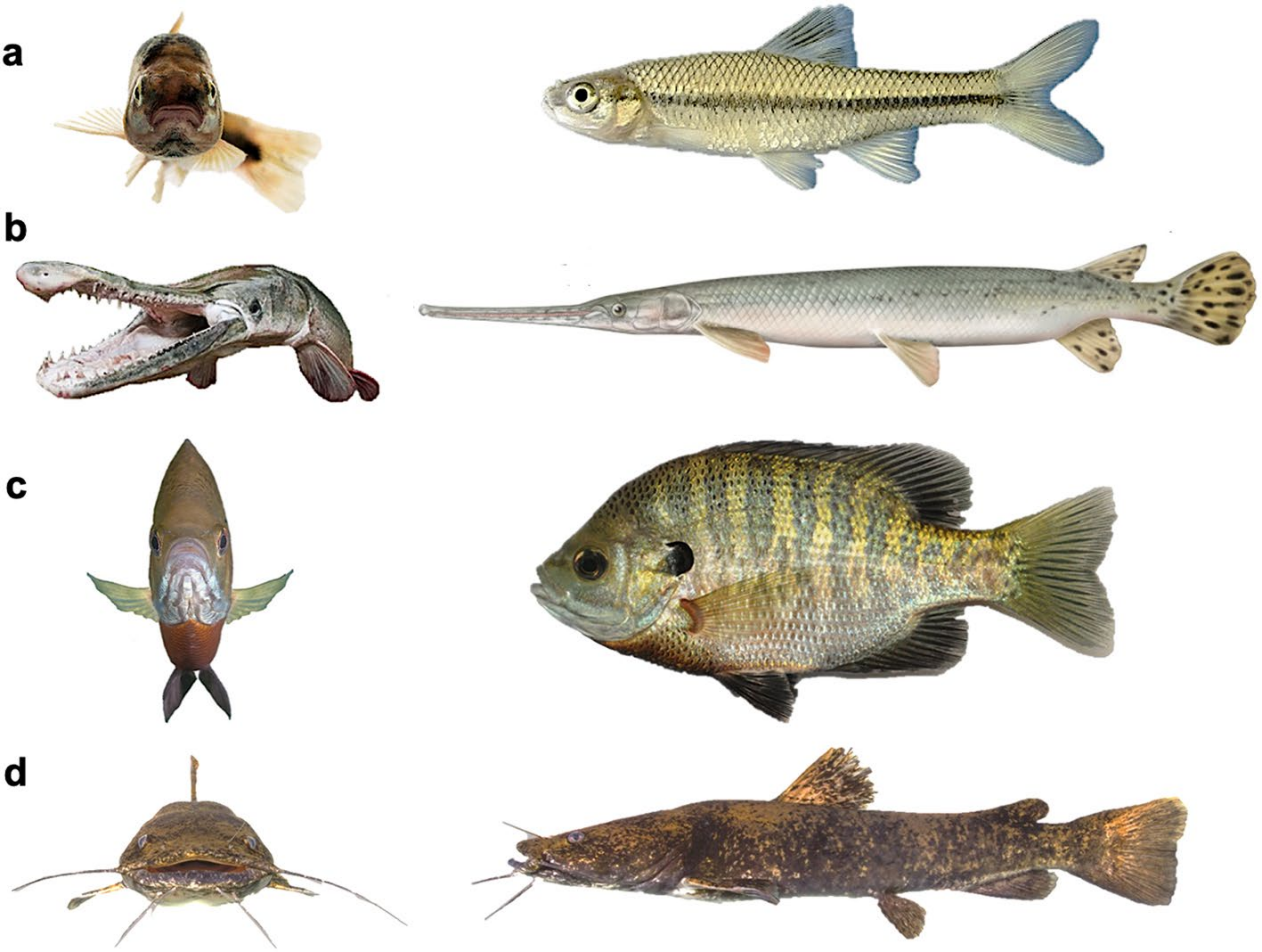
Common LMB fish species with exemplar body shapes. (**a**) The Pugnose Minnow *Opsopoeodus emiliae* (Leuciscidae), to 6.4 cm standard length (SL). (**b**) Longnose Gar *Lepisosteus osseus* (Lepisosteidae), to 122 cm SL. (**c**) Bluegill *Lepomis macrochirus* (Centrarchidae), to 30 cm SL. (**d**) Flathead Catfish *Pylodictis olivaris* (Ictaluridae), to 155 cm SL.

Specimens used in this analysis of LMB fish body shape range in size from 19 mm in the Least Killifish (*Heterandria formosa,* Poeciliidae) to 987 mm TL in the Bull Shark (*Carcharhinus leucas,* Carcharhinidae). The mean adult body shape of LMB fishes is calculated to be most closely approximated by the Pugnose Minnow (*Opsopoeodus emiliae,* Leuciscidae), which possesses a fusiform body, a relatively short snout and small head, an approximately mid-body dorsal-fin insertion, ventrally-positioned pectoral fins, and posteriorly-positioned pelvic and anal fins (Fig. 2A). Fishes with an elongate snout or rostrum (e.g., lepisosteid gars, *Polyodon* paddlefish, *Strongylura* needlefishes; Fig. 2B) exhibit trait values that are furthest from the overall centroid, as estimated by the sum of the absolute value of all the weighted PC values. Fishes with extreme positive PC1 and PC2 values exhibit a laterally-compressed and dorsoventrally deep body shape, laterally-positioned pectoral fins, and anteriorly positioned pelvic fins (Fig. 2C) contrasting with dorsoventrally compressed species such as catfishes (Fig. 2D).

The 3D eigenvectors for all landmarks used in the body-shape analysis of LMB fishes are arranged by relative magnitude in Table 2. PC1 represents 51.1% of the shape variance seen in LMB fishes, and is dominated by landmarks associated with the dorsal, pectoral, and pelvic fin positions, which together explain 62.8% of variance in PC1. PC2 explaining 15.9% of the total variance is largely affected by the positions of the dorsal, anal, and pelvic fin insertions, which together explain 56.6% of PC2. PC3 explains 13.4% and is mostly affected by dorsal, anal, and pelvic fin positions, which total to 58.5% of PC3. PC4 explains 6.4% of the total variance and is dominated by snout length and pectoral-fin position, which together account for 60.9% of PC3. PC5 explains 5.1% of the total variance, and is largely affected by snout length, pectoral fin position, and anterior anal-fin insertion, totaling 60.9% of PC5. PC6 is also dominated by pectoral-fin position and snout length, totaling 55.6% of PC6 (see Table S1).

**Table 2:** Summary of eigenvector magnitudes for all landmarks used in the geometric morphometric analysis of LMB fish body shape.

Landmark	L#	PC 1	PC 2	PC 3	PC 4	PC 5	PC 6	Sum	Norm (%)
DF anterior insertion	7	0.312	0.093	0.09	0.002	0.011	0.007	0.515	18.44
Left P2	4	0.189	0.067	0.036	0.015	0.014	0.005	0.326	11.67
Right P2	10	0.19	0.066	0.035	0.015	0.014	0.005	0.325	11.64
AF anterior insertion	5	0.101	0.041	0.058	0.047	0.018	0.007	0.272	9.74
Left P1	3	0.136	0.028	0.028	0.018	0.020	0.012	0.242	8.66
Right P1	9	0.135	0.029	0.028	0.017	0.021	0.011	0.241	8.63
Tip snout	1	0.121	0.019	0.031	0.031	0.027	0.012	0.241	8.63
Left Hypural	6	0.099	0.033	0.026	0.014	0.007	0.003	0.182	6.52
Right Hypural	11	0.099	0.033	0.026	0.014	0.007	0.002	0.181	6.48
Right eye	8	0.074	0.032	0.009	0.007	0.008	0.006	0.136	4.87
Left eye	2	0.074	0.031	0.008	0.005	0.008	0.006	0.132	4.73

Note positions of median and paired fin insertions dominate all the PCs. Landmark positions are shown in Methods. Data presented for top six PCs representing 95% of the total variance. Landmarks ranked by their total (summed) eigenvector magnitudes. L#, landmark number; Norm, normalized.

**Figure 3:**
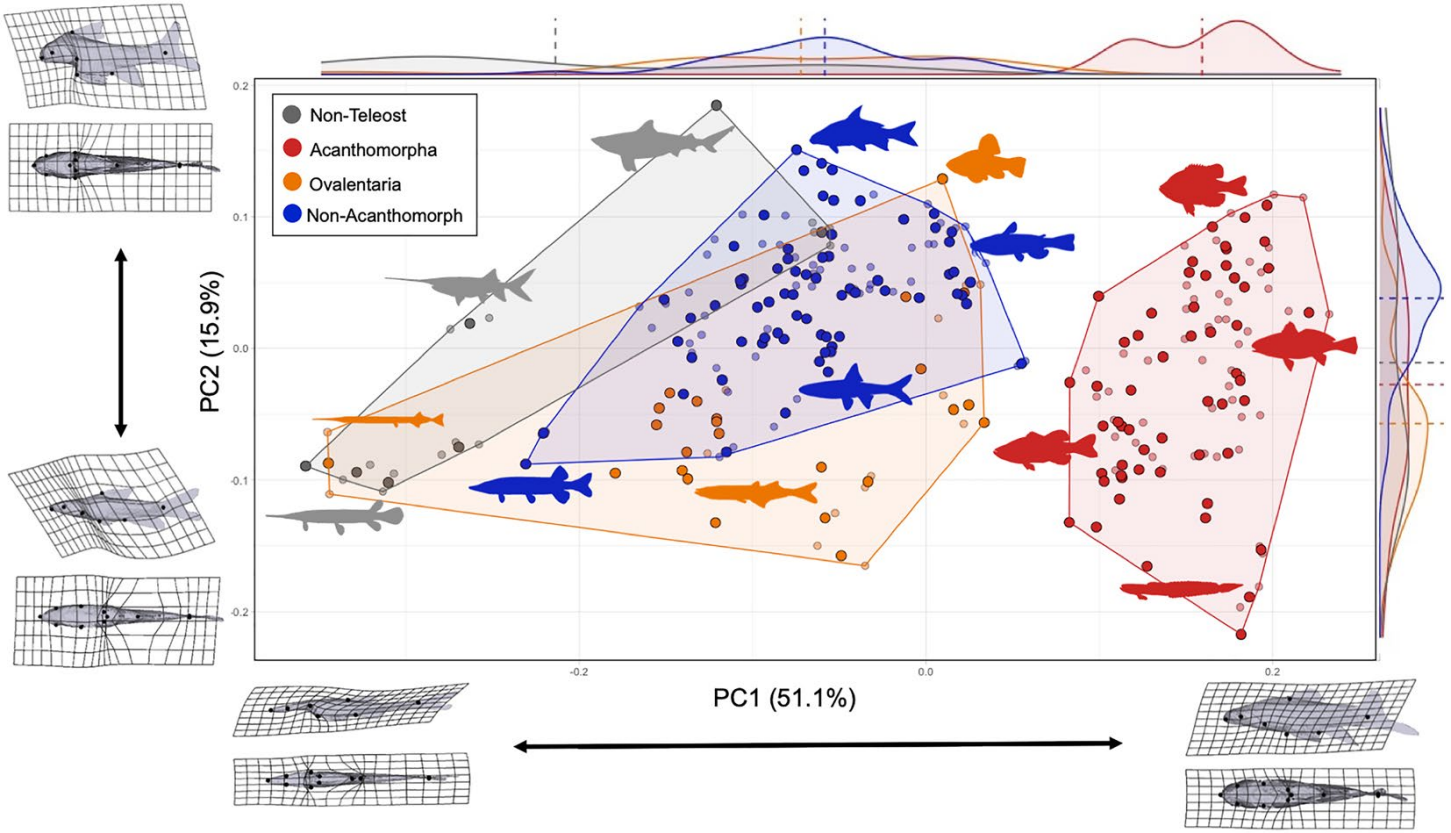
Morphospace of LMB fishes from PCA of 3D landmark data. Data for 232 specimens (smaller circles) in 166 species (larger circles) representing 37 family-level clades. PC1 on horizontal axis, PC2 on vertical axis. Relative frequency histograms along PC axes with mean values as dashed lines. Deformation grids illustrating extreme PC values for each axis in lateral and ventral views. Color scheme as in Fig. 1.

The PCA resulted in a morphospace of adult body shape in LMB fishes (Fig. 3). In these bivariate plots of PC1 against PCs 2–6, each specimen and species mean is represented by small and large circles, respectively. The taxa in Figs. 1 and 3 are color-coded to represent nested clades in the analysis of morphospace occupancy and are not reciprocally monophyletic. We group the LMB taxa into these four nested categories to study the evolution of shape disparity in an explicitly phylogenetic context. The groups of taxa indicated by colored polygons are not proposed as units of phylogeny or grades of phenotypic evolution, but rather as nested taxa occupying portions of the LMB morphospace at different time intervals. We note that these hypotheses are limited by taxonomic representation of the extant LMB fishes, which can be reevaluated with more complete taxonomic and phenotypic sampling including examination of fossil fishes through time. Frequency histograms summarizing the sample density for each PC axis are provided along the top and right margins.

Within the LMB fish morphospace, PC1 represents variance along the anteroposterior axis, from fishes with an elongate, slender body and posteriorly positioned dorsal and pelvic fins (e.g., lepisosteid gars, belonid needlefish, esocid pikes), to species with more anteriorly-positioned dorsal and pelvic fins and wide range of body depths (Fig. 3). Species in this fauna with low PC1 values are polyphyletic, representing four phylogenetically disparate clades, while species with high PC1 values are only represented by acanthomorph teleosts, an extraordinarily diverse clade including about one-fifth of the world's modern species of vertebrates (> 14,000 species). The density histograms indicate that fishes with high PC1 values dominate the LMB fish morphospace due to the high species-richness of centrarchid sunfishes and percid darters.

Within this morphospace, PC2 represents variance along the dorsoventral axis in lateral view, ranging from fishes with a deeper to a more slender body shape in lateral profile, with greatest variance in the dorsoventral position of the dorsal, pelvic, and anal fin insertions. Fishes with deepest body shapes (at least one third as deep as long) have evolved multiple times in teleosts^72^ and are represented by at least five independent clades among LMB fishes. Despite being the derived condition, the frequency histogram indicates that the modal condition of LMB fishes is to have a relatively deep body, as observed in catostomid suckers, and cyprinodontid killifishes.

**Figure 4:**
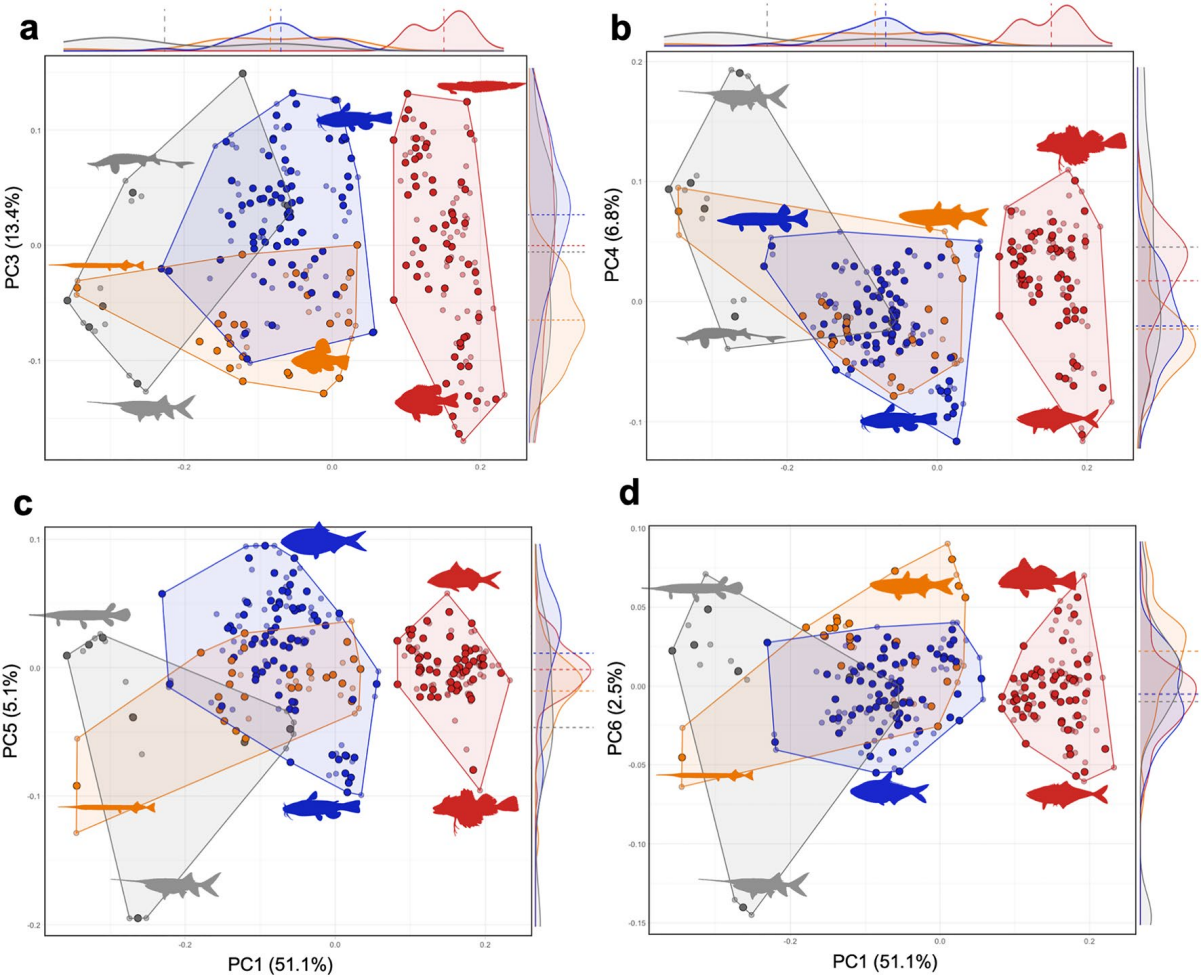
Morphospace analysis of LMB fishes from 3D landmark data in PCs 3–6. Data for 232 specimens (smaller circles) in 166 species (larger circles) representing 40 family-level clades present in the LMB fish fauna. PC1 on horizontal axis, PC3–6 on vertical axes. Relative frequency histograms along PC axes with mean values as dashed lines. Colors as in Fig. 3. (**a**) PC1 versus PC3. (**b**) PC1 versus PC4. (**c**) PC1 versus PC5. (**d**) PC1 versus PC6.

Within the LMB fish morphospace, PC3 represents variance along the dorsoventral axis in frontal view, ranging from fishes with a more vertically compressed and wider body shape (e.g., the Bull Shark *Carcharhinus leucas*, acipenserid sturgeons, catostomid suckers, ictalurid catfishes, and the Violet Goby *Gobioides broussonnetii*), to deeper and more laterally compressed bodies (cyprinodontid killifishes, centrarchid sunfishes; Fig. 4A). The frequency histogram indicates a bimodal distribution, with about half of the species exhibiting high PC3 values and the other half exhibiting low PC3 values. Both extreme values of PC3 have been evolved multiple times.

In our analysis, PCs 4–6 are all strongly influenced by the extreme body shape of the Paddlefish *Polyodon*, which is an outlier (Fig. 4B,C,D). PC4 is most strongly affected by landmarks associated with the relative size of the head as compared to the rest of the body, with larger PC4 values representing larger head size. Large relative head size is present in multiple distinct LMB fish clades, and the frequency distribution is positively skewed with more fishes having smaller heads (see Table S2). PC5 represents dorsoventral body compression with stronger compression of the head. Relatively few species exhibit strongly negative PC5 values, being restricted to belonid needlefish, *Polyodon* paddlefish, ictalurid catfishes, and a triglid Sea Robin. PC6 represents expansion of the midbody compared to head and tail observed in midwater pelagic taxa (*Polyodon* paddlefish, *Dorosoma* shads, *Caranx* jacks). Relatively few species exhibit strong PC6 values, which is restricted to marine-derived taxa and *Polyodon* paddlefish.

**Figure 5:**
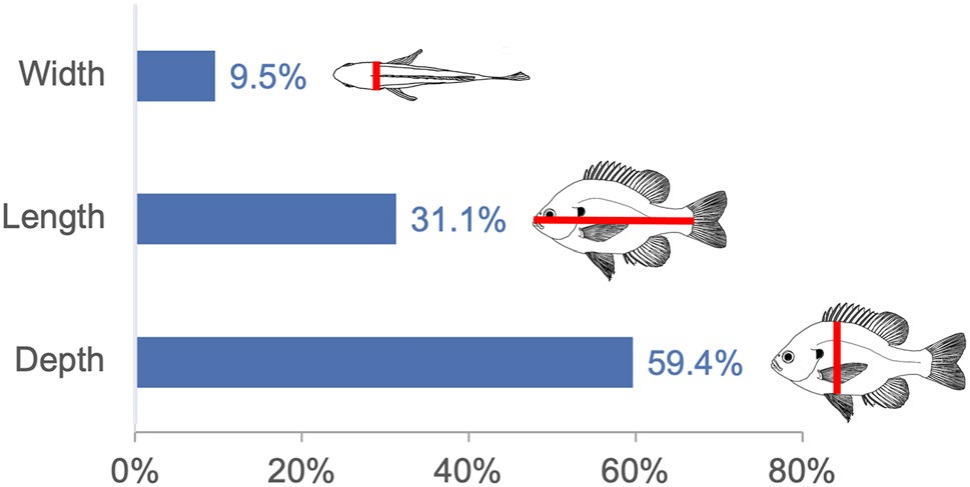
Summed variance of landmark deformations in each of the three spatial dimensions. Note the greater variance in dorsoventral (depth) than anteroposterior (length) or mediolateral (width) landmark positions. Note also the relative magnitude of variance among spatial dimensions differs from the absolute size of these dimensions; i.e. length > depth > width.

Body shape disparity in LMB fishes is dominated by variance in body depth, which represents 59.4% of the total variance, and is strongly represented in all the top PCs (Fig. 5). Landmarks strongly influenced by body depth include dorsal, pelvic, and anal fin insertions. Differences in landmark positions along the long body axis constitute 31.1% of the total variance, and include important functional traits associated with the position of dorsal and anal-fin insertions, tip of snout, and caudal margin of hypural plates. Differences in landmarks associated with body width represent only 9.5% of the total variance, and are the most constrained among the three spatial dimensions of body shape variance.

## Discussion

The mean body shape of LMB fishes is fusiform, with midbody depth c. 25% standard length, a head length c. 30% standard length, and dorsal and pelvic fins vertically aligned near maximum body depth near midbody. Thus although LMB fishes represent a fraction of global fish diversity, the mean body shape of this fauna closely resembles the estimated plesiomorphic body shape of teleost fishes^72,73^. This notable similarity may result from the disproportionate representation of Cypriniformes with 69 species that compose 28% of the LMB fauna, which retain a plesiomorphic teleostean body shape.

The major dimensions of body-shape disparity in the LMB fish fauna are also similar to those of other fish faunas worldwide, both marine and freshwater^36^, but with some interesting differences. The first three PCs (PC1–3), encompassing 80.4% of total body shape variance, represent aspects of shape evolution with widespread convergence within and among the major fish clades^72^. The next three PCs (PC4–6), encompassing 14.4% of the variance, represent shapes of younger and less diverse clades, with *Polyodon* paddlefish exhibiting the extreme phenotype in all three of these PCs.

The colored polygons in Figs. 1 and 3 represent nested clades that highlight aspects of the phylogenetic structure of the morphospace occupancy. These groups are not reciprocally monophyletic, and they do not represent evolutionary or phylogenetic groups per se. Rather these three artificial and one natural groups draw attention to portions of the larger tree that exhibit substantial changes in aspects of body shape. In this morphospace, PC1 represents variance from taxa with elongate snouts and slender bodies to short snouts and deep bodies, with the highest PC1 values observed only in non-ovalentarian acanthomorphs. This is notable because Acanthomorpha, representing c. 40% of all teleost species, includes Ovalentaria with some species that have elongate snouts and slender bodies (e.g. belonid needlefishes).

The predominance of depth over length in the magnitude of LMB fish body shape variance differs from that reported in other studies of fish body shape (Table 1), where body elongation dominates PC1^36,37^. One possible reason for this discrepancy is taxonomic composition, with marine faunas dominated by acanthomorphs and freshwater faunas by ostariophysan teleosts. Another possible reason is different measurement data, with our study based on 3D landmarks of major body regions and fin positions and previously published studies based on point-to-point distance measurements and ratios and 2D landmarks^57,58^. The predominance of depth over other dimensions strongly differs from non-GM studies based on linear measurements, for which the data were not subjected to Procrustes superimposition. This is not to say that GM is superior, but that different methods can give different qualitative results.

### PCs as growth fields

Phenotypic disparity among members of a clade or assemblage may accrue from multiple ecological and evolutionary drivers^76–78^. These processes include phenotypic divergence within a region due to drift or selection^79^, dispersal (including establishment) of taxa from other biotas^80,81^, and the regional extirpation of taxa that may reduce disparity or result in phenotypic discontinuities^82,83^. Because phenotypic evolution arises from changes in the developmental program that descendants inherit from their ancestors^84^, body shape differences among species ultimately arise from changes in developmental growth fields^85–87^. Under this evo-devo perspective, each PC of body-shape variance may be hypothesized to represent a distinct, putative, homologous, phylogenetic growth field shared by the taxa that vary along this axis^86,87^. Under this hypothesis, each PC is a phylogenetic character that can potentially evolve due to changes in gene expression affecting developmental growth fields^49,50,88^.

All the top six PCs in the morphospace of Figs. 2 and 3, accounting for more than 95% of the total body-shape variance in the LMB fauna, represent ancient and rare phylogenetic events. Each of these statistically distinct aspects of body-shape variance (PCs 1–6) are derived from evolutionary transformations that occurred millions of years ago in one or a few clades. To summarize the results described above, PC1 largely represents changes in dorsal, pectoral, and pelvic fin positions of acanthomorph teleosts, PC2 and PC3 changes in dorsal, anal, and pelvic fin insertions among members of each of the four nested clades depicted as colored polygons in Fig. 1, PC4 changes in snout length and pectoral-fin position among members of these four nested clades, PC5 changes in snout length, pectoral-fin position, and anterior anal-fin insertion of these same clades, and PC6 changes in pectoral-fin position and snout length of these same clades. A major similarity underlying variance in all of these PCs is that each evolved only one to several times deep in the fish phylogeny of Fig. 1. Each of these PCs is here hypothesized to represent changes in homologous growth fields derived from one or a few phylogenetic events.

Shape disparity data from LMB fishes does not indicate evolution along lines of least evolutionary resistance^89^. The greatest aspect of ontogenetic shape change from larvae to adult in most fishes is negative head allometry^90,91^. Yet differential growth of the head and post-cranial body regions is negligible on PCs 1–3 which constitute the great majority of the total shape variance, and loading most strongly on PC4 with about 6.8% of the variance (Fig. 4B). In other words, the most important axes of shape variance within and among species are not aligned. Shape change associated with the growth of individual fishes is highly plastic among LMB species, whereas shape changes observed at the family level and above are highly conserved, representing millions to tens of millions of years of phenotypic conservatism.

### Assessing fish shape disparity

Results of this study using 3D GM provide a more complete understanding of fish body-shape disparity than can be achieved using 2D GM (Fig. 5) due to the inclusion of an additional dimension of information. The body shape of several morphologically diverse and ecologically important LMB fish clades (e.g., acipenserid sturgeons, ictalurid catfishes) is strongly compressed dorsoventrally, such that PC3 represents 13.4% of the total shape variance in the whole fauna. 3D GM also allows quantitative comparisons of body shape among other fish faunas worldwide, allowing investigators to identify gaps or other constraints in body-shape morphospaces^50^. Comparative studies can also quantify the contribution of taxa with extreme body shapes to overall body shape disparity (e.g., anguilliform, gymnotiform, and synbranchiform eel-shaped taxa; syngnathiform pipefishes, seahorses, and seadragons). Identifying landmarks on the body margins of these taxa is however challenging, as most lack one or more of the homologous fins used to anchor the body-shape morphospace of LMB fishes.

Results of this study also suggest caution in using overall body shape as a proxy for functional diversity in freshwater fishes^92^. While certain portions of the LMB fish morphospace are occupied by taxa with characteristic locomotory modes (e.g., acceleration predators, elongate burrowing gobies, pelagic cruisers), most of the morphospace is occupied by species with a range of behavioral traits and ecological functions. In fact, many LMB fish species and families broadly overlap in the morphospace (Figs. 3 and 4). In other words, while extreme body shapes are often associated with distinct functions, most fishes do not have extreme shapes, and the shapes of most fishes are used in a variety of functional contexts. When evaluating the role of the fins and mouth positions as predictors of habitat and diet in fishes, it is almost always important to have information on internal anatomy, physiology and behavior^8,93,94^. Overall body shape must therefore be considered a relatively coarse measure of locomotory function for most fishes^67^.

### Undergraduate research education experiences

3D photogrammetry provides a relatively easy, inexpensive, and engaging entry into meaningful museum collection-based research for undergraduate researchers. Museum collections provide priceless repositories of past and present organisms, yet most collections are not broadly understood or appreciated by the public^74^. Engaging undergraduate students in meaningful projects using these resources provides experiences in specimen care and curation, data acquisition, analysis, and presentation, and an appreciation for collections that will last after graduation. Many undergraduate students are eager to participate in mentored research opportunities but are intimidated by steep learning curves and high levels of required knowledge and technical skills, which discourages broad participation of all groups in STEM^75^. The flexibility and easy workflow of our method for 3D photogrammetry gives confidence to students who can use their own cell phone cameras to do real science.

A majority of the 3D models of LMB fishes used in this analysis were generated by undergraduate students from the University of Louisiana at Lafayette as a part of Course-embedded Undergraduate Research Experiences (CURES) in an upper-level Ichthyology class during Fall semester 2022. Many of these students were highly engaged and showed great enthusiasm for the project and later joined the lab as research assistants as part of Mentored Undergraduate Research Experiences (MURES). We report the highest level of engagement and enthusiasm for the required research-based class project in the 18 years that the course has been taught by the senior author. Four of the authors of this paper are undergraduate students who invested substantially in the development of the methods, data acquisition, and analysis.

## Materials and Methods

### Sampling and specimen selection

The LMB fauna excludes coastal marine fishes that have never been collected inland and temperate zone fishes present in the upper Mississippi. We included freshwater and brackish water species listed in the most recent faunal compilation^25^, several of which represent primarily marine taxa rarely collected in freshwaters (e.g., carangid jacks, scombrid mackerel, triglid sea robins), which diverge strongly from the shape of the core freshwater fauna and are therefore not expected to strongly influence the functional disparity of LMB fishes.

3D models of body shape were generated using 3D photogrammetry of 232 preserved fish specimens representing 166 of 245 (68%) species, 37 of 45 (82%) families, and 24 of 28 (86%) orders of the LMB fish fauna (see Table S3). All materials are housed at the University of Louisiana at Lafayette Ichthyology Teaching Collection (ULL), Louisiana State University Museum of Natural Science (LSUMZ), Auburn University Museum of Natural History (AUM) and the Florida Museum of Natural History (UF) collections. No live specimens were used in this study.

### 3D model generation and editing

**Figure 6:**
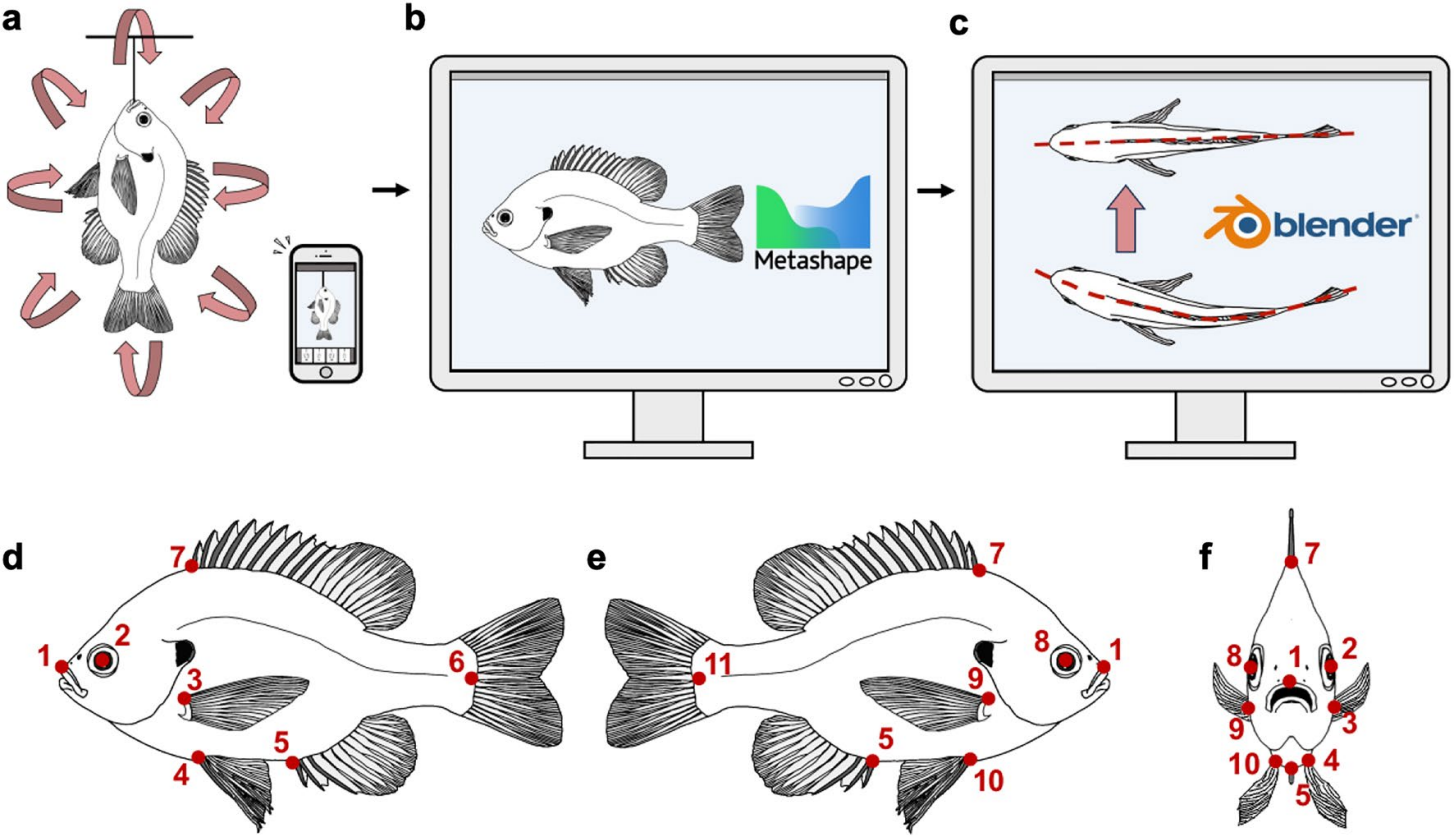
Digitalization of biological specimens for downstream multivariate analyses using 3D photogrammetry. (**a**) Specimen is prepared and suspended at an appropriate height to allow for 360° access and photography by the researcher in an evenly lit location. 150–600 overlapping photographs are taken from every angle using modern cell phone cameras; however, we note that more expensive cameras may provide 3D models with higher levels of surface detail. (**b**) Digital photographs of specimen are loaded into Agisoft Metashape for 3D reconstruction. 3D models were exported as .obj files with .jpg textures. (**c**) 3D models were then individually loaded into Blender to clean and carefully straighten while still maintaining their overall natural shape. The preservation and long-term storage of wet biological specimens often results in the specimen becoming permanently bent or twisted, confounding any GM analysis of shape, and thus requiring a method of unbending. Unbent and cleaned 3D models were then exported from Blender to be used in the GM analysis. (**d**) Landmark scheme of 11 homologous landmarks used in the GM analysis in left lateral view. (**e**) Landmark scheme in right lateral view. (**f**) Landmark scheme in anterior view to convey 3D nature of landmarks.

A majority of the 3D models used in this study were generated by undergraduate research assistants beginning in Fall 2022 through Summer 2023 semesters. The exterior surface of each specimen was lightly dried to reduce reflective surfaces which prove difficult for 3D photogrammetric model generation in subsequent steps. Small, complex, transparent, and reflective surfaces and features are difficult to reconstruct. Prepared specimens were individually suspended by the mouth or gill opening from the ceiling with a length of pliable wire in an environment with bright, even lighting^95^. Small specimens with a total length (TL) < 60 mm and those too large to safely suspend required special adaptations to the methods (camera macro settings, etc.). Approximately 150–600 overlapping photographs were taken of each specimen from multiple angles and distances to achieve full overlapping coverage of the external features of the specimen in each photoset and capture small details of the body surface. Varying the distance from the camera to the specimen improves the ability of the software to reconstruct a 3D model and render the detailed surface textures. The number of photos in each photoset was largely a function of the size of each specimen, with larger specimens requiring more photographs for complete external coverage. Photosets were loaded into the software Metashape (Agisoft; https://www.agisoft.com) to reconstruct textured 3D models of each specimen using default alignment settings and following standard instructions from the developer available on their website (https://www.agisoft.com/support/tutorials/). Models were exported in .obj (Wavefront) format with accompanying texture files in .jpg and material files in .mtl formats. Model post-processing, including cropping and smoothing, was done in Blender 3.3.1 (https://www.blender.org/). Models of specimens that were permanently bent or curved due to preservation or long-term storage were made straight while maintaining their overall shape by using the rotate (R) and grab (G) functions while in Edit Mode. Scale is set in Blender by providing a scale factor calculated from a known distance between two points on each model. The processed models were then re-exported as new .obj files that were used in the 3D analyses (Fig. 6).

### Landmarking and 3D analyses

The landmarks used in this 3D GM study are points along the dorsal, ventral, and lateral surfaces of the head and post-cranial body indicated by major skeletal discontinuities (Fig. 6). These landmarks are widely used to assess fish body shape in phylogenetic and functional studies to demarcate homologous body regions^96,97^. Evidence for the homology of these landmarks comes from ultrastructural, embryological, and topological aspects of similarity along with phylogenetic congruence with other traits^98–102^. Because the post-cranial landmarks are based on fin insertions, several species lacking homologous landmarks of median or paired fins were excluded from the morphospace analysis (i.e., *Syngnathus* pipefish, anguilliform eels, and *Trinectes* flatfishes; purple taxa in Fig. 1).

We assessed body-shape variance using fixed 3D landmarks of prominent features demarcating boundaries of major body regions (Fig. 6). We did not use semilandmarks or pseudolandmarks that cover the surface of the 3D models (e.g., 3D landmark meshes) because they were not needed to assess the major aspects of body shape disparity under investigation, and because these methods are highly sensitive to preservational artifacts that are methodologically demanding to control. These artifacts include the variable orientation and condition of fins, barbels, and other epidermal protrusions (e.g. odontodes, cirri) in preserved specimens, and unnaturally distended or sunken abdominal cavities in specimens of soft-bodied species. Correcting these artifacts would require considerable time and effort while not serving the purpose of assessing the major features of body shape which are the target of this study.

Models in .obj format were individually loaded into 3D Slicer^103^ and texture files in .jpg format were applied to the models using the Texture Model module from the SlicerIGT extension^104^. Landmarks were then placed on each 3D mesh using Slicer’s fiducial function and exported as individual .fcsv landmark files. All landmark files were then loaded into Slicer and a Generalized Procrustes Analysis (GPA) and Principal Component Analysis (PCA) were performed in the SlicerMorph GPA module^105^. GPA translates all 3D landmark configurations to the same centroid, scales the landmark configurations to the same centroid size (root summed squared distance of the landmarks from their centroid), and rotates the landmark configurations to minimize the summed squared differences between the configurations and their sample average. Results were loaded into R using the ‘SlicerMorphR’ (https://github.com/SlicerMorph/SlicerMorphR) package. Visualization and analysis of the morphospace including the generation of warp/deformation grids was done using the ‘geomorph’ package^106^. The mean adult body shape of LMB fishes was approximated using the ‘mshape’ function. Colored polygons circumscribing groups in the PCA morphospace are minimum convex hulls calculated in R using the ‘ggplot2’ package^86,87,107^.

### Phylogeny

The phylogeny of Fig. 1 depicts a widely-used, time-calibrated tree of fish families based on the topology of Rabosky et al^108^. Although this phylogeny has known incongruencies with aspects of the primary literature at the species level, it is largely concordant with more recently published fish phylogenies^109^ and classifications^110^ at the family level. This tree is presented to highlight the phylogenetic distribution and structure of LMB fishes among the global fish fauna^108^. The tree was imported and vizualized in R using the ‘ape’^111^ and ‘phytools’ packages^112^.

### Eigenvector analysis

Eigenvectors were exported from R and used to analyze the contribution of each landmark to each PC, and to the total morphospace. In the analysis of eigenvector loadings, the directional values, which are either positive or negative, were weighted by the percent variance explained by each PC. The magnitude of the 3D vector of each landmark was calculated as the square root of the sum of the squares of the three eigenvectors (x, y, z). Note these 3D vectors are all positive since the value of each individual component is squared. Results are presented as normalized values for each landmark as percentage of total variance (Table S1).

## Supplementary materials

Supplementary Materials including TS1-3, R scripts, and all data necessary to replicate the analysis have been published alongside this manuscript as Supplementary Material and/or have been made available at the Dryad repository (https://doi.org/10.5061/dryad.n2z34tn2t).

### Supplementary Information

Supplementary Information 1.

Supplementary Tables.

## Data Availability

All data needed to evaluate the conclusions in the paper are present in the paper and/or the Supplementary Materials. A supplementary table (TS2) and all data and R code to reproduce the presented analyses are available at 10.5061/dryad.n2z34tn2t.
