# Supplementary material for: Phylogenetic structure of body shape in a diverse inland ichthyofauna
Source: Sci Rep. 2023 Nov 25;13:20758. doi: 10.1038/s41598-023-48086-5 (PMC10676429; doi:10.1038/s41598-023-48086-5)
Supplement: Supplementary file 2 — Supplementary Tables. [file 41598_2023_48086_MOESM2_ESM.pdf]

Supplementary Materials for

Phylogenetic structure of body shape in a diverse inland ichthyofauna

Kevin T. Torgersen *et al.*

*Corresponding author. Email: kevintorgersen@gmail.com

This PDF file includes:

Tables S1, S3

Other Supplementary Materials for this manuscript include the following:

Table S2
Data S1, S2
R Scripts

Table S1. Eigenvectors for PCA of 3D GM of LMB fish fauna. Both raw and values weighted by contribution of each PC are reported.

**Raw eigenvectors**											
Dimension	Landmark	PC 1	PC 2	PC 3	PC 4	PC 5	PC 6	PC 7	PC 8	PC 9	PC 10
X	1.X	-0.233	-0.003	0.224	0.441	0.472	-0.371	0.065	-0.032	0.297	-0.040
Y	1.Y	-0.041	-0.118	0.066	-0.086	-0.221	-0.275	-0.403	0.193	0.098	-0.564
Z	1.Z	0.002	0.003	0.010	-0.005	-0.005	0.006	0.028	0.001	0.009	0.039
X	2.X	-0.134	-0.054	0.017	-0.038	-0.058	0.100	-0.230	-0.028	-0.468	0.077
Y	2.Y	0.045	-0.147	0.007	0.043	-0.089	-0.069	0.055	0.249	0.025	0.436
Z	2.Z	-0.030	0.114	-0.055	0.055	-0.125	-0.196	0.020	-0.208	-0.044	0.241
X	3.X	-0.249	-0.030	-0.203	-0.253	-0.220	0.150	0.152	-0.061	0.340	-0.027
Y	3.Y	0.062	0.155	-0.029	0.023	0.178	0.276	-0.245	-0.382	0.131	0.042
Z	3.Z	-0.067	0.085	-0.016	0.047	-0.270	-0.372	0.032	-0.292	-0.039	-0.008
X	4.X	0.334	0.394	0.059	0.223	-0.190	0.093	0.107	0.121	-0.014	-0.105
Y	4.Y	-0.157	0.143	-0.258	-0.005	0.176	-0.051	0.234	0.010	-0.223	-0.144
Z	4.Z	-0.011	0.051	0.039	-0.029	-0.096	-0.155	-0.124	-0.158	-0.081	-0.034
X	5.X	0.124	0.212	0.345	-0.684	0.347	-0.253	0.081	0.036	-0.043	0.086
Y	5.Y	-0.155	0.143	-0.259	0.060	0.064	-0.062	0.107	0.174	-0.141	-0.119
Z	5.Z	-0.001	0.005	-0.007	-0.002	-0.018	0.018	0.017	0.001	0.012	0.010
X	6.X	-0.187	-0.204	0.112	0.197	-0.007	0.096	-0.061	0.003	-0.009	0.006
Y	6.Y	0.050	0.042	0.156	-0.044	-0.130	-0.045	-0.155	0.131	0.151	0.050
Z	6.Z	-0.003	0.007	0.005	-0.008	-0.002	-0.018	0.008	-0.016	-0.009	0.004
X	7.X	0.579	-0.415	-0.524	-0.020	0.161	-0.236	-0.091	-0.110	0.039	-0.028
Y	7.Y	0.196	-0.415	0.418	-0.006	-0.129	0.140	0.510	-0.336	-0.145	-0.208
Z	7.Z	0.001	0.002	-0.005	0.012	-0.003	0.000	-0.004	0.012	0.015	0.040
X	8.X	-0.135	-0.055	0.011	-0.041	-0.056	0.095	-0.236	-0.014	-0.468	0.080
Y	8.Y	0.045	-0.149	0.015	0.052	-0.076	-0.100	0.059	0.253	0.014	0.440
Z	8.Z	0.029	-0.119	0.062	-0.070	0.132	0.207	-0.022	0.194	0.038	-0.276
X	9.X	-0.248	-0.027	-0.210	-0.244	-0.244	0.133	0.155	-0.042	0.341	0.022
Y	9.Y	0.063	0.156	-0.023	0.021	0.188	0.261	-0.268	-0.402	0.150	0.099
Z	9.Z	0.063	-0.091	0.008	-0.035	0.273	0.325	-0.077	0.294	0.022	-0.030
X	10.X	0.337	0.384	0.059	0.222	-0.194	0.106	0.118	0.130	-0.001	-0.083
Y	10.Y	-0.156	0.144	-0.252	-0.011	0.166	-0.042	0.272	0.008	-0.196	-0.126
Z	10.Z	0.011	-0.046	-0.048	0.038	0.101	0.174	0.126	0.167	0.074	0.031
X	11.X	-0.188	-0.201	0.111	0.197	-0.011	0.087	-0.059	-0.003	-0.012	0.012
Y	11.Y	0.047	0.044	0.157	-0.045	-0.127	-0.033	-0.165	0.103	0.136	0.093
Z	11.Z	0.005	-0.010	0.008	-0.002	0.012	0.012	-0.006	0.005	0.003	-0.018

Weighted eigenvectors		PC 1	PC 2	PC 3	PC 4	PC 5	PC 6	PC 7	PC 8	PC 9	PC 10
% contribution of each PC		51.1%	15.9%	13.4%	6.8%	5.1%	2.5%	1.4%	1.0%	0.9%	0.05%
X	1.X	-0.119	0.000	0.030	0.030	0.024	-0.009	0.001	0.000	0.003	0.000
Y	1.Y	-0.021	-0.019	0.009	-0.006	-0.011	-0.007	-0.006	0.002	0.001	-0.003
Z	1.Z	0.001	0.000	0.001	0.000	0.000	0.000	0.000	0.000	0.000	0.000
X	2.X	-0.069	-0.009	0.002	-0.003	-0.003	0.002	-0.003	0.000	-0.004	0.000
Y	2.Y	0.023	-0.023	0.001	0.003	-0.005	-0.002	0.001	0.002	0.000	0.002
Z	2.Z	-0.015	0.018	-0.007	0.004	-0.006	-0.005	0.000	-0.002	0.000	0.001
X	3.X	-0.127	-0.005	-0.027	-0.017	-0.011	0.004	0.002	-0.001	0.003	0.000
Y	3.Y	0.031	0.025	-0.004	0.002	0.009	0.007	-0.003	-0.004	0.001	0.000
Z	3.Z	-0.034	0.013	-0.002	0.003	-0.014	-0.009	0.000	-0.003	0.000	0.000
X	4.X	0.171	0.063	0.008	0.015	-0.010	0.002	0.002	0.001	0.000	-0.001
Y	4.Y	-0.080	0.023	-0.035	0.000	0.009	-0.001	0.003	0.000	-0.002	-0.001
Z	4.Z	-0.006	0.008	0.005	-0.002	-0.005	-0.004	-0.002	-0.002	-0.001	0.000
X	5.X	0.064	0.034	0.046	-0.046	0.018	-0.006	0.001	0.000	0.000	0.000
Y	5.Y	-0.079	0.023	-0.035	0.004	0.003	-0.002	0.001	0.002	-0.001	-0.001
Z	5.Z	-0.001	0.001	-0.001	0.000	-0.001	0.000	0.000	0.000	0.000	0.000
X	6.X	-0.096	-0.032	0.015	0.013	0.000	0.002	-0.001	0.000	0.000	0.000
Y	6.Y	0.026	0.007	0.021	-0.003	-0.007	-0.001	-0.002	0.001	0.001	0.000
Z	6.Z	-0.001	0.001	0.001	-0.001	0.000	0.000	0.000	0.000	0.000	0.000
X	7.X	0.296	-0.066	-0.070	-0.001	0.008	-0.006	-0.001	-0.001	0.000	0.000
Y	7.Y	0.100	-0.066	0.056	0.000	-0.007	0.004	0.007	-0.003	-0.001	-0.001
Z	7.Z	0.001	0.000	-0.001	0.001	0.000	0.000	0.000	0.000	0.000	0.000
X	8.X	-0.069	-0.009	0.001	-0.003	-0.003	0.002	-0.003	0.000	-0.004	0.000
Y	8.Y	0.023	-0.024	0.002	0.004	-0.004	-0.003	0.001	0.003	0.000	0.002
Z	8.Z	0.015	-0.019	0.008	-0.005	0.007	0.005	0.000	0.002	0.000	-0.001
X	9.X	-0.127	-0.004	-0.028	-0.017	-0.012	0.003	0.002	0.000	0.003	0.000
Y	9.Y	0.032	0.025	-0.003	0.001	0.010	0.007	-0.004	-0.004	0.001	0.000
Z	9.Z	0.032	-0.015	0.001	-0.002	0.014	0.008	-0.001	0.003	0.000	0.000
X	10.X	0.172	0.061	0.008	0.015	-0.010	0.003	0.002	0.001	0.000	0.000
Y	10.Y	-0.079	0.023	-0.034	-0.001	0.008	-0.001	0.004	0.000	-0.002	-0.001
Z	10.Z	0.006	-0.007	-0.006	0.003	0.005	0.004	0.002	0.002	0.001	0.000
X	11.X	-0.096	-0.032	0.015	0.013	-0.001	0.002	-0.001	0.000	0.000	0.000
Y	11.Y	0.024	0.007	0.021	-0.003	-0.006	-0.001	-0.002	0.001	0.001	0.000
Z	11.Z	0.003	-0.002	0.001	0.000	0.001	0.000	0.000	0.000	0.000	0.000

Table S2. (separate file) Component scores for 26 PCs for all 232 specimen models used in the analysis of LMB body shape.

Table S3. Museum collection specimens used for 3D photogrammetry and the 3D GM analysis. TL, total length in mm; N, number of specimens used.

Order	Family	Species	TL (mm)	N	Catalog #
Acanthuriformes	Sciaenidae	*Aplodinotus grunniens*	101–280	2	ULL 24
Acanthuriformes	Sciaenidae	*Bairdiella chrysoura*	117	1	ULL 49
Acanthuriformes	Sciaenidae	*Cynoscion arenarius*	99	1	ULL 6070
Acanthuriformes	Sciaenidae	*Cynoscion nebulosus*	127–225	3	ULL 26, ULL 41, UL 6053
Acanthuriformes	Sciaenidae	*Leiostomus xanthurus*	78	1	ULL 6042
Acanthuriformes	Sciaenidae	*Micropogonias undulatus*	127–132	3	ULL 23
Acanthuriformes	Sciaenidae	*Pogonias cromis*	220–270	2	ULL 18, ULL 51
Acanthuriformes	Sciaenidae	*Sciaenops ocellatus*	65–220	2	ULL 25, ULL 39
Acipenseriformes	Acipenseridae	*Scaphirhynchus platorynchus*	452–605	3	ULL 6098, ULL 6099, LSUMZ 21101
Acipenseriformes	Polyodontidae	*Polyodon spathula*	245–257	2	ULL 6089
Amiiformes	Amiidae	*Amia calva*	74–160	4	UF 119608, LSUMZ 10736
Atheriniformes	Atherinopsidae	*Labidesthes sicculus*	70–83	2	ULL 6058
Atheriniformes	Atherinopsidae	*Membras martinica*	106	1	LSUMZ 20000
Atheriniformes	Atherinopsidae	*Menidia audens*	70	1	ULL 6045
Atheriniformes	Atherinopsidae	*Menidia beryllina*	66–88	2	ULL 6051, ULL 6054
Beloniformes	Belonidae	*Strongylura marina*	131–312	2	ULL 70, ULL 6088
Carangiformes	Carangidae	*Caranx hippos*	135–190	2	LSUMZ 15752, ULL 6073
Carangiformes	Carangidae	*Oligoplites saurus*	124–146	2	LSUMZ 2687, ULL 378
Carcharrhiniformes	Carcharinidae	*Carcharhinus leucas*	987	1	LSUMZ Uncat.
Centrarchiformes	Centrarchidae	*Ambloplites ariommus*	133–134	2	LSUMZ 18411
Centrarchiformes	Centrarchidae	*Centrarchus macropterus*	136–177	2	LSUMZ 943, LSUMZ 12609
Centrarchiformes	Centrarchidae	*Elassoma zonatum*	38	1	ULL 6049
Centrarchiformes	Centrarchidae	*Lepomis auritus*	97	1	ULL 2718
Centrarchiformes	Centrarchidae	*Lepomis cyanellus*	95	1	ULL 6087
Centrarchiformes	Centrarchidae	*Lepomis gulosus*	99	1	ULL 6055
Centrarchiformes	Centrarchidae	*Lepomis macrochirus*	131–183	2	ULL 6083
Centrarchiformes	Centrarchidae	*Lepomis marginatus*	65	1	ULL 6028
Centrarchiformes	Centrarchidae	*Lepomis megalotis*	116–182	2	ULL 1409, ULL 6069
Centrarchiformes	Centrarchidae	*Lepomis microlophus*	121	1	ULL 6056
Centrarchiformes	Centrarchidae	*Lepomis symmetricus*	74	1	ULL 681

Centrarchiformes	Centrarchidae	*Micropterus dolomieu*	185	1	ULL 6041
Centrarchiformes	Centrarchidae	*Micropterus punctulatus*	68	1	ULL 1497
Centrarchiformes	Centrarchidae	*Micropterus salmoides*	105–130	2	ULL 2688, ULL 6078
Centrarchiformes	Centrarchidae	*Pomoxis annularis*	182	1	ULL 1498
Centrarchiformes	Centrarchidae	*Pomoxis nigromaculatus*	135–191	2	ULL 94
Centrarchiformes	Moronidae	*Morone chrysops*	278	1	ULL 6076
Centrarchiformes	Moronidae	*Morone mississippiensis*	122	1	ULL 6063
Centrarchiformes	Moronidae	*Morone saxatilis*	161	1	ULL 6034
Cichliformes	Cichlidae	*Herichthys cyanoguttatus*	109–150	2	ULL 6062
Clupeiformes	Clupeidae	*Alosa alabamae*	140	1	ULL 6074
Clupeiformes	Clupeidae	*Alosa chrysochloris*	234–360	2	ULL 124, ULL 6033
Clupeiformes	Clupeidae	*Brevoortia patronus*	106–250	2	ULL 6032, ULL 6090
Clupeiformes	Clupeidae	*Dorosoma cepedianum*	112–114	2	ULL 6082
Clupeiformes	Clupeidae	*Dorosoma petenense*	97	1	ULL 6044
Clupeiformes	Engraulidae	*Anchoa mitchilli*	76–87	2	ULL 398, LSUMZ 2163
Cypriniformes	Catostomidae	*Carpiodes carpio*	163	1	LSUMZ 12942
Cypriniformes	Catostomidae	*Carpiodes cyprinus*	207	1	LSUMZ 3210
Cypriniformes	Catostomidae	*Carpiodes velifer*	258	1	LSUMZ 13048
Cypriniformes	Catostomidae	*Cycleptus meridionalis*	479	1	LSUMZ 17649
Cypriniformes	Catostomidae	*Erimyzon sucetta*	61	1	ULL 5097
Cypriniformes	Catostomidae	*Erimyzon tenuis*	163–248	2	ULL 3142, LSUMZ 10085
Cypriniformes	Catostomidae	*Hypentelium nigricans*	114–159	2	ULL 3144
Cypriniformes	Catostomidae	*Ictiobus bubalus*	153	1	ULL 6084
Cypriniformes	Catostomidae	*Ictiobus cyprinellus*	156	1	LSUMZ 317
Cypriniformes	Catostomidae	*Ictiobus niger*	97	1	LSUMZ 18244
Cypriniformes	Catostomidae	*Minytrema melanops*	107	1	LSUMZ 20964
Cypriniformes	Catostomidae	*Moxostoma poecilurum*	161–220	2	ULL 6075, UF 132238
Cypriniformes	Cyprinidae	*Campostoma anomalum*	104	1	LSUMZ 18236
Cypriniformes	Cyprinidae	*Carassius auratus*	174	1	ULL 159
Cypriniformes	Cyprinidae	*Cyprinella camura*	84	1	LSUMZ 11398
Cypriniformes	Cyprinidae	*Cyprinella venusta*	79–182	3	ULL 1498, ULL 2427, ULL 2428
Cypriniformes	Cyprinidae	*Cyprinella whipplei*	105	1	LSUMZ 1067
Cypriniformes	Cyprinidae	*Cyprinus carpio*	96–238	2	ULL 161, ULL 725
Cypriniformes	Cyprinidae	*Hybognathus nuchalis*	99	1	LSUMZ 20682
Cypriniformes	Cyprinidae	*Hybopsis winchelli*	70	1	LSUMZ 11346
Cypriniformes	Cyprinidae	*Hypognathus hayi*	70	1	ULL 6035
Cypriniformes	Cyprinidae	*Luxilus chrysocephalus*	128	1	LSUMZ 18260
Cypriniformes	Cyprinidae	*Lythrurus roseipinnis*	55	1	LSUMZ 12772
Cypriniformes	Cyprinidae	*Lythurus umbratilis*	70	1	ULL 6093
Cypriniformes	Cyprinidae	*Macrhybopsis storeriana*	121	1	LSUMZ 18482

Cypriniformes	Cyprinidae	*Nocomis leptocephalus*	136	1	LSUMZ 20329
Cypriniformes	Cyprinidae	*Notemigonus crysoleucas*	55	1	ULL 6071
Cypriniformes	Cyprinidae	*Notropis amplamala*	78	1	LSUMZ 3199
Cypriniformes	Cyprinidae	*Notropis atherinoides*	66	1	ULL 6040
Cypriniformes	Cyprinidae	*Notropis atrocaudalis*	52	1	ULL 6043
Cypriniformes	Cyprinidae	*Notropis blennius*	60	1	ULL 6064
Cypriniformes	Cyprinidae	*Notropis boops*	64	1	LSUMZ 21148
Cypriniformes	Cyprinidae	*Notropis longirostris*	59	1	LSUMZ 12507
Cypriniformes	Cyprinidae	Notropis maculatus	63	1	LSUMZ 21114
Cypriniformes	Cyprinidae	*Notropis potteri*	96–97	3	ULL 6080
Cypriniformes	Cyprinidae	*Notropis sabinae*	42	1	ULL 3608
Cypriniformes	Cyprinidae	*Notropis shumardi*	62	1	LSUMZ 20399
Cypriniformes	Cyprinidae	*Notropis texanus*	107	1	ULL 6057
Cypriniformes	Cyprinidae	*Notropis volucellus*	57	1	ULL 6039
Cypriniformes	Cyprinidae	*Notropis wickliffi*	62	1	LSUMZ 15379
Cypriniformes	Cyprinidae	*Opsopoeodus emiliae*	63	1	ULL 3222
Cypriniformes	Cyprinidae	*Phenacobius mirabilis*	94	1	LSUMZ 17748
Cypriniformes	Cyprinidae	*Pimephales notatus*	75	1	LSUMZ 18130
Cypriniformes	Cyprinidae	*Pimephales vigilax*	80	1	LSUMZ 18358
Cypriniformes	Cyprinidae	*Pteronotropis signipinnis*	41	1	LSUMZ 4208
Cypriniformes	Cyprinidae	*Semotilus atromaculatus*	109	1	LSUMZ 3728
Cypriniformes	Xenocyprididae	*Ctenopharyngodon idella*	189–214	3	ULL 5864, LSUMZ 2807
Cyprinodontiformes	Cyprinodontidae	*Cyprinodon variegatus*	56	1	ULL 3434
Cyprinodontiformes	Fundulidae	*Fundulus blairae*	64	1	LSUMZ 12081
Cyprinodontiformes	Fundulidae	*Fundulus catenatus*	94	1	LSUMZ 17741
Cyprinodontiformes	Fundulidae	*Fundulus chrysotus*	58–60	2	ULL 6047, ULL 6095
Cyprinodontiformes	Fundulidae	*Fundulus euryzonus*	71	1	ULL 6037
Cyprinodontiformes	Fundulidae	*Fundulus grandis*	72	1	ULL 6094
Cyprinodontiformes	Fundulidae	*Fundulus majalis*	82	1	ULL 6036
Cyprinodontiformes	Fundulidae	*Fundulus notatus*	62	1	ULL 6096
Cyprinodontiformes	Fundulidae	*Fundulus nottii*	52	1	ULL 6038
Cyprinodontiformes	Fundulidae	*Fundulus olivaceus*	76	1	ULL 6029
Cyprinodontiformes	Fundulidae	*Fundulus pulvereus*	48	1	ULL 1135
Cyprinodontiformes	Fundulidae	*Fundulus similis*	83	1	ULL 6085
Cyprinodontiformes	Fundulidae	*Fundulus xenicus*	34	1	ULL 6086
Cyprinodontiformes	Fundulidae	*Lucania parva*	41	1	ULL 1413
Cyprinodontiformes	Poeciliiidae	*Gambusia affinis*	38	1	ULL 683
Cyprinodontiformes	Poeciliiidae	*Heterandria formosa*	19	1	ULL 6046
Cyprinodontiformes	Poeciliiidae	*Poecilia latipinna*	48–60	4	ULL 289, ULL 290, ULL 6092
Elopiformes	Elopidae	*Elops saurus*	109–201	3	ULL 6059, LSUMZ 20015

Esociformes	Esocidae	*Esox americanus*	236	1	ULL 3781
Esociformes	Esocidae	*Esox niger*	92–97	2	ULL 174
Esociformes	Umbridae	*Umbra limi*	71–74	2	ULL 241
Gobiiformes	Eleotridae	*Dormitator maculatus*	96–105	2	ULL 165
Gobiiformes	Oxudercidae	*Ctenogobius shufeldti*	67	1	ULL 236
Gobiiformes	Oxudercidae	*Evorthodus lyricus*	67	1	ULL 5784
Gobiiformes	Oxudercidae	*Gobioides broussonnetii*	238	1	ULL 769
Gobiiformes	Oxudercidae	*Gobionellus oceanicus*	160–167	2	ULL 6065
Hiodontiformes	Hiodontidae	*Hiodon alosoides*	82–166	2	AUM 37830, LSUMZ teaching collection uncat.
Hiodontiformes	Hiodontidae	*Hiodon tergisus*	87–99	2	AUM 41881
Lepisosteiformes	Lepisosteidae	*Atractosteus spatula*	331–620	2	ULL 6100, ULL 6101
Lepisosteiformes	Lepisosteidae	*Lepisosteus oculatus*	239–250	2	ULL 6102, ULL 6103
Lepisosteiformes	Lepisosteidae	*Lepisosteus osseus*	272	1	LSUMZ 15827
Mugiliformes	Mugilidae	*Dajaus monticola*	253	1	LSUMZ 14651
Mugiliformes	Mugilidae	*Mugil cephalus*	98–152	2	ULL 516, ULL 6072
Mugiliformes	Mugilidae	*Mugil curema*	121	1	LSUMZ 19950
Osmeriformes	Osmeridae	*Osmerus mordax*	102–171	3	AUM 18867
Perciformes	Gerreidae	*Eucinostomus argenteus*	112–121	2	ULL 183, LSUMZ 14263
Perciformes	Lutjanidae	*Lutjanus griseus*	98–241	3	ULL 6077, LSUMZ 15950, LSUMZ 19406
Perciformes	Percidae	*Ammocrypta beanii*	64	1	ULL 1115
Perciformes	Percidae	*Ammocrypta vivax*	58	1	ULL 2885
Perciformes	Percidae	*Crystallaria asprella*	99	1	LSUMZ 1583
Perciformes	Percidae	*Etheostoma caeruleum*	52	1	ULL 2229
Perciformes	Percidae	*Etheostoma histrio*	44	1	ULL 6068
Perciformes	Percidae	*Etheostoma parvipinne*	60	1	ULL 203
Perciformes	Percidae	*Etheostoma stigmaeum*	49	1	LSUMZ 11832
Perciformes	Percidae	*Etheostoma swaini*	44	1	ULL 6067
Perciformes	Percidae	*Etheostoma whipplei*	51	1	ULL 6048
Perciformes	Percidae	*Percina caprodes*	83–124	2	ULL 198, ULL 1247
Perciformes	Percidae	*Percina copelandi*	48	1	LSUMZ 11658
Perciformes	Percidae	*Percina macrolepida*	113	1	LSUMZ 15996
Perciformes	Percidae	*Percina maculata*	71	1	ULL 197
Perciformes	Percidae	*Percina nigrofasciata*	75–104	2	ULL 199, ULL 4652
Perciformes	Percidae	*Percina sciera*	70	1	ULL 210
Perciformes	Percidae	*Percina uranidea*	55	1	ULL 2947
Perciformes	Percidae	*Sander canadensis*	82–281	2	LSUMZ 1140, LSUMZ 1461
Perciformes	Sparidae	*Archosargus probatocephalus*	118–228	2	LSUMZ 2164, LSUMZ 2690
Percopsiformes	Aphredoderidae	*Aphredoderus sayanus*	55–57	2	ULL 224
Scorpaeniformes	Triglidae	*Prionotus tribulus*	92–97	2	ULL 337, ULL 338
Scrombriformes	Scombridae	*Scomberomorus maculatus*	98–140	2	ULL 6030, ULL 6061

Siluriformes	Ariidae	*Ariopsis felis*	276	1	ULL 776
Siluriformes	Ariidae	*Bagre marinus*	146–243	2	ULL 6052, LSUMZ 4465
Siluriformes	Ictaluridae	*Ameiurus melas*	70–236	5	ULL 251, ULL 6031, ULL 6081
Siluriformes	Ictaluridae	*Ameiurus natalis*	107	1	LSUMZ 19124
Siluriformes	Ictaluridae	*Ameiurus nebulosus*	92	1	LSUMZ 18245
Siluriformes	Ictaluridae	*Ictalurus furcatus*	167	1	ULL 4561
Siluriformes	Ictaluridae	*Ictalurus punctatus*	115	1	ULL 6079
Siluriformes	Ictaluridae	*Noturus funebris*	83	1	LSUMZ 17561
Siluriformes	Ictaluridae	*Noturus gyrinus*	59	1	ULL 6060
Siluriformes	Ictaluridae	*Noturus leptacanthus*	114	1	LSUMZ 1757
Siluriformes	Ictaluridae	*Noturus miurus*	66	1	LSUMZ 11158
Siluriformes	Ictaluridae	*Pylodictis olivaris*	181	1	LSUMZ 19134

Data S1. (separate file) 3D landmark data files in .fcsv format for 232 3D models used in the 3D GM analysis.

Data S2. (separate file) Output files from SlicerMorph GPA module to be processed in R.
